# Anomaly Detection and Repairing for Improving Air Quality Monitoring

**DOI:** 10.3390/s23020640

**Published:** 2023-01-06

**Authors:** Federica Rollo, Chiara Bachechi, Laura Po

**Affiliations:** “Enzo Ferrari” Engineering Department, University of Modena and Reggio Emilia, 41121 Modena, Italy

**Keywords:** low-cost sensors, air quality sensors, air quality monitoring, anomaly detection, anomaly repairing, multivariate time series

## Abstract

Clean air in cities improves our health and overall quality of life and helps fight climate change and preserve our environment. High-resolution measures of pollutants’ concentrations can support the identification of urban areas with poor air quality and raise citizens’ awareness while encouraging more sustainable behaviors. Recent advances in Internet of Things (IoT) technology have led to extensive use of low-cost air quality sensors for hyper-local air quality monitoring. As a result, public administrations and citizens increasingly rely on information obtained from sensors to make decisions in their daily lives and mitigate pollution effects. Unfortunately, in most sensing applications, sensors are known to be error-prone. Thanks to Artificial Intelligence (AI) technologies, it is possible to devise computationally efficient methods that can automatically pinpoint anomalies in those data streams in real time. In order to enhance the reliability of air quality sensing applications, we believe that it is highly important to set up a data-cleaning process. In this work, we propose AIrSense, a novel AI-based framework for obtaining reliable pollutant concentrations from raw data collected by a network of low-cost sensors. It enacts an anomaly detection and repairing procedure on raw measurements before applying the calibration model, which converts raw measurements to concentration measurements of gasses. There are very few studies of anomaly detection in raw air quality sensor data (millivolts). Our approach is the first that proposes to detect and repair anomalies in raw data before they are calibrated by considering the temporal sequence of the measurements and the correlations between different sensor features. If at least some previous measurements are available and not anomalous, it trains a model and uses the prediction to repair the observations; otherwise, it exploits the previous observation. Firstly, a majority voting system based on three different algorithms detects anomalies in raw data. Then, anomalies are repaired to avoid missing values in the measurement time series. In the end, the calibration model provides the pollutant concentrations. Experiments conducted on a real dataset of 12,000 observations produced by 12 low-cost sensors demonstrated the importance of the data-cleaning process in improving calibration algorithms’ performances.

## 1. Introduction

Air pollution is currently the most significant environmental risk to human health, and European citizens perceive it as the second-biggest environmental concern after climate change [1]. Monitoring air quality is of primary importance to encourage more sustainable lifestyles and plan corrective actions [2]. The essential tool for monitoring air quality is a network of devices [3,4], usually organized in a wireless sensor network supported by Internet of Things (IoT) technology [5]. Among the AQ devices, low-cost sensors are now acknowledged as a unique means to gather high spatio-temporal air quality data through dense monitoring networks thanks to their economic feasibility [6,7,8]. However, low-cost sensors have many limitations: electrochemical gas sensors, very often used in air quality monitoring, indeed, have high unit-to-unit variability and suffer from drift components such as aging and concept drift, depending on the calibration approach [9,10,11]. Therefore, the need for a hyper-local sensing system able to monitor pollutants’ variations in urban areas collides with the poor quality of measurements generated by low-cost air quality sensors. This kind of sensor generally is not able to measure a pollutant concentration directly; thus, they require a calibration process performed in the same environment in which they will use to convert the raw measurements into pollutant concentrations. The quality of the calibration process is of major importance in order to generate reliable air quality data. The presence of anomalies in raw data negatively influences the performances of data-driven calibration models [12]. However, removing anomalies increases the number of missing values in the multivariate time series of raw measurements. Since these data-driven models are often based on more than one feature, one for each of the measured variables, the presence of a missing value or an outlier in one variable can compromise the prediction of the entire observation. In the air quality monitoring context, anomaly detection, data cleaning, and repairing methodologies have usually been applied on the calibrated observations as post-processing techniques [13]. In our approach, the anomaly detection and repairing are applied to the raw sensors readings (millivolts) captured by low-cost sensors before the application of the calibration model. Only in a few studies have the raw data been preprocessed to remove possible outliers, and generally this has been done using statistical methods. In [14], for example, anomalies were removed using a filter based on the computation of a local polynomial (R LOESS function) and the median absolute deviation (MAD) between this polynomial and the measurements within a floating window. In [15], the outliers were detected using a DBSCAN algorithm that slightly improved the correlation between the target gas data and the sensors’ data. Our approach goes beyond mere statistical distributions and takes into account different characteristics of air quality sensor data: the time dependence of the observation and the correlations among pollutants and environmental measurements.

The AIrSense framework is a comprehensive solution to deal with low-cost sensor data, from the collection of raw observations to the generation of reliable pollutants concentrations. Thanks to low-cost sensors and Artificial Intelligence (AI) techniques, we can identify anomalies in the raw data measured by the sensors, remove them, and repair the missing values. This data-cleaning pre-processing of the raw data allows the calibration model to run on cleaned information, thereby improving the precision and reliability of the air quality (AQ) monitoring system. AIrSense is able to provide location-specific, real-time environmental data and actionable insights to public administrations and citizens to mitigate the effects of various threats from environmental factors, such as air pollution.

The contributions of this paper are as follows:

- We propose and implemented a comprehensive solution for large-scale AQ sensing systems: AIrSense. The proposed framework is simple, effective, and capable of detecting and repairing anomalous data and is available as open source software (https://github.com/ChiaraBachechi/AQAnomalyDetectionFramework (accessed on 30 December 2022)).
- We performed anomaly detection through the application of three algorithms that take into account different characteristics of air quality sensors: a univariate anomaly detection (the sliding window anomaly detection), a multivariate anomaly detection algorithm (the forgetting factor iterative data capture anomaly detection), and an algorithm based on the dependencies among pollutants measurements and the measurements of temperature and humidity (the temperature and humidity based anomaly detection).
- We validated the results of the anomaly detection model following two approaches: a supervised evaluation with the help of environmental experts and unsupervised validation through the comparison with a well-known anomaly detection algorithm considering two synthetic datasets: one with extreme outliers, and the other with variance outliers.
- The effects of anomaly detection and repairing have been evaluated on the calibration models that estimate the pollutant concentration values; experiments based on a real-world dataset demonstrated that the RMSE is significantly reduced by introducing anomaly detection before calibration, and the accuracy was improved by adding the repairing procedure.

The outline of the paper is the following. Section 2 summarizes the current state of the art, and Section 3 describes the AIrSense framework. In Section 4, the algorithms for anomaly detection are described in detail, and the results of their application are discussed and validated. The anomaly repairing technique is disclosed in Section 5, along with its application and results. Section 6 is devoted to the description of three experiments and their evaluation, and in Section 7, conclusions are reported.

## 2. Related Work

**Anomaly detection.** Detection of anomalies in time series has received a considerable attention in the literature [12,16,17,18,19,20,21]. With the diffusion and advancement of IoT technologies, the rapid processing of sensor data streams challenges traditional data-handling solutions and asks for new approaches. The environment where IoT devices are developed makes them vulnerable to failure and malfunction, leading to the generation of unusual and erroneous data [22,23,24,25,26]. On univariate or multivariate time series, anomaly detection is mainly performed through clustering or distance-based techniques [27,28], prediction [29,30,31], statistical approaches [32,33], deep learning methodologies using autoencoders [18,34,35], and neural networks [36,37,38]. In environmental datasets, the occurrence of high concentrations of an unusual pollutant may indicate air quality problems. Thus, a critical understanding of the behavior of anomalies is increasingly becoming very important for air pollution investigations. Several techniques have been explored in order to detect outliers in gases or particle observations through functional analysis [39], the probability finite-state-automata-based algorithm, statistical methods [40,41,42], or combined methods [43].

**Anomaly repairing.** Removing detected anomalies creates some missing values that, especially in the case of time-series data, bring the necessity of repairing techniques to fill in the missing values. Repairing or gap filling allows one to patch the holes generated after the removal of anomalies [44,45]. In [46], the problem of repairing dirty time-series data, given the labeled truth of some data points, was studied, and it was demonstrated with several experiments that adapting existing anomaly detection techniques to anomaly repairing is inconsistent with the minimum change principle of data repairing. Thus, the authors proposed an iterative minimum repairing algorithm, performing one minimum repair in each iteration. In the context of geo-distributed sensor networks [47,48], anomalies can be repaired by exploiting non-anomalous data measured by the sensors in nearby spatial locations. This solution can be applied to pollutants’ concentrations but does not apply to low-cost air quality sensor raw measurements, since, as described in Section 3, the chemical cells of different sensors are not comparable. For this reason, the correlations among close sensors cannot be exploited to identify anomalies. This, instead, can be done for other kind of sensors, such as traffic sensors [49,50]. Other solutions for gap filling are the “imputeTS” package [51], which provides a collection of algorithms and tools especially tailored to repair univariate time series and the Kalman filter [52], which is able to fill the gaps by estimating past, present, and future values.

## 3. AIrSense Framework

**Figure 1:**
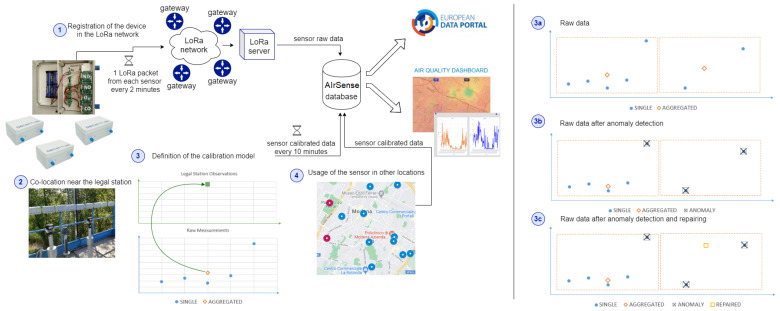
The production of raw data, calibration, and export in the AIrSense framework (on the **left**) and the anomaly detection and repairing procedure on raw data (on the **right**).

The AIrSense framework is in charge of collecting the data produced by low-cost sensors, finding anomalies and repairing them by defining the calibration model for each sensor, and exploiting the model for providing gas concentrations, starting with the raw measurements. Figure 1 reports the entire AQ monitoring process. We start with the deployment of a sensor network, where each low-cost device needs to be registered in the network (point 1, in the figure).

**Data collection.** In our case, we set up a long-range wide area network (LoRaWAN) [53], a media access control protocol widely used in smart city applications. The networks employ gateways, i.e., antennas, that receive broadcast messages from the AQ devices and forward them to the LoRa server where the messages are interpreted. The raw data are extracted and sent to the AIrSense database, which we developed following the approach described in [54]. For each sensor, the calibration was performed by co-locating the sensors close to legal stations for a certain period (point 2 in figure), called the calibration period (or co-location period). During this period, we register the status of the device in “calibration” mode. During the co-location, the values of pollutants concentrations measured by the legal station and the raw observations of the low-cost sensors are collected and aggregated every 10 min to generate a comparable dataset (point 3 in figure). The calibration period is used to train a calibration model for each sensor (i.e., for each gas of each device). Once the calibration model has been generated, the device can be moved anywhere in the city, and the pollutant concentrations are calculated in real-time from raw observations by exploiting the calibration model (point 4 in figure). During this period, we register the status of the device in “running” mode. The lifetime of a sensor might be 3–5 years; however, its monitoring performance degrades over time. Periodically, the devices are moved back close to the legal station for a new calibration period, thereby re-calibrating the model and improving the performance (from point 4 to point 2 in figure). All the data produced are collected and stored in a database. Moreover, raw and calibrated data are available as open data on the Emilia Romagna regional data portal (https://dati.emilia-romagna.it/ (accessed on 30 December 2022)) and also on the National and European data portals; the hourly data of the legal stations are available on the ARPAE data portal (https://dati.arpae.it/dataset/qualita-dell-aria-rete-di-monitoraggio (accessed on 30 December 2022)); the datasets discussed in Section 6 are available as open data (https://drive.google.com/drive/folders/1LqZSVXA_2A1Hk_7fk9UwDOYEda-J6qvG (accessed on 30 December 2022)) and displayed in a dashboard [55,56].

**Air quality sensors.** Point 1 in Figure 1 shows the exterior and interior of a Decentlab sensor (https://www.decentlab.com/products/air-quality-station-no2-no-co-ox-for-lorawan (accessed on 30 December 2022)). These are the AQ devices we mainly employed in the TRAFAIR project [57]. Inside the box, there is a sensor for air temperature and humidity, and four AQ sensors for NO, NO_2_, CO, and O_3_. Each device provides two raw measures in millivolt (mV) for each pollutant through the working (we) electrode and the auxiliary (aux) electrode (also called channels), in addition to the air temperature (°C), humidity (%), and battery voltage. Each raw measurement is a couple of variable and value (e.g., (NO${}_{aux}$, 1050 mV)). In a specific moment, a device captures a raw observation that is the set of 13 raw measurements (e.g., {(temperature, 13 °C), (humidity, 45%), (NO${}_{aux}$, 1050 mV), (NO${}_{we}$, 3450 mV)}).

**Criticalities.** It may happen that during the lifetime of an AQ device, some problems arise, such as dirt or spiders entering the device; the device getting wet, falling, or being damaged by vandalism; etc. In such cases, the environmental engineers responsible for the installation and maintenance of the devices report the problems by setting the status of the device to “broken”. Other more serious issues are malfunctioning or the breakdown of a cell. This will require a replacement of the cell itself. During this period, the device’s status is set to “off-line”. The cell replacement obviously will also have consequences for the calibration model that must be recalculated, since every single cell is different and requires an ad hoc calibration model. Even if sensors are located in the same position, it is not possible to compare their raw data, since the chemical cells inside each sensor are unique and can measure very different millivolt values for the same values of pollutant concentrations in the air. For this reason, every single cell needs a specific calibration model, and the anomaly detection procedure cannot be based on neighboring sensors. A customized dashboard has been developed to allow environmental engineers to keep track of all status changes and maintenance operations [58].

**Data aggregation.** The AIrSense framework starts with the collection of raw data from AQ sensors. Raw measurements are thereafter aggregated every 10 min, as displayed in Figure 1(3a), and a mean value is calculated in a 10 min interval. By performing anomaly detection directly on raw data, anomalies are excluded from the raw measurements before the aggregation process (Figure 1(3b)). Therefore, the mean value will not be affected by these outliers (as reported on the left side of Figure 1(3b)), and the calibration model will be trained on cleaned data, reducing the error on the estimated pollutant concentration.

**Data repairing.** In some cases, all the raw values in the 10 min interval may be labeled as anomalies (as reported on the right side of Figure 1(3b)); therefore, a gap is created in the time series of aggregated values. In order to avoid these gaps, the AIrSense framework allows repairing by predicting the missing values based on the previous raw observations (as reported on the right side of Figure 1(3c)).

**Calibration.** The calibration model translates a raw observation into a calibrated observation that provides the pollutant concentrations. These sensor calibrated data are created in real-time every 10 min, starting from the raw measurements in that interval. In the literature, there are several examples of long short-term memory (LSTM) employed for AQ prediction [59,60]. LSTM [61] is a recurrent neural network suitable for time-series data, and can take into account an arbitrarily long past sequence to predict future values. We used LSTM to generate the pollutant concentrations from the raw observations.

## 4. Anomaly Detection

Anomaly detection is performed through the application of a majority voting system (MV) on the raw measurements. MV combines three algorithms: sliding window anomaly detection (Section 4.1), the forgetting factor iterative data capture anomaly detection (Section 4.2), and the temperature and humidity-based anomaly detection (Section 4.3). For each gas, the algorithms consider the two channels of raw measurements (e.g., NO${}_{aux}$ and NO${}_{we}$) separately; if at least one of them is anomalous, then the gas measurement is an anomaly. Each algorithm has the same weight in the ensemble method and detects anomalies for each pollutant individually; thus, if at least two out of three algorithms classify the pollutant measurement as anomalous, that measurement will be considered an outlier. The three selected algorithms exploit different strategies and are based on diverse correlations among data. Therefore, their combination allows classifying anomalies with higher confidence than applying only one algorithm.

### 4.1. Sliding Window Anomaly Detection: SWAD

The sliding window anomaly detection algorithm (SWAD) is a combination of the differences-based algorithm and the interquartile range (IQR). This algorithm is an example of univariate anomaly detection.

The differences-based algorithm evaluates the difference between two consecutive values. The “difference threshold” corresponds to the maximum variation allowed in a fixed period. Given the threshold ϵ, if $|x_{t}-x_{t-1}|>$ϵ, then $x_{t}$ is classified as anomalous. Intuitively, the variation in observed values in a few minutes is expected to be small, since pollutant measurements do not change rapidly. Defining the optimal value of the threshold is tricky; it could be set to a constant by domain experts or calculated according to the input data by statistical methods. The weak aspect of this method is the incapacity of finding consecutive anomalies, since the difference between two anomalies is assumed to be small. For this reason, this algorithm is combined with the study of the interquartile range (IQR).

The interquartile range (IQR) analyzes the distribution of the raw measurements and is defined as the difference between the third quartile (q3) and the first quartile (q1). By using these values, the upper bound and the lower bound of the acceptable measurements range is found as follows:

(1) lowerbound=q1−q∗IQR

(2) upperbound=q3+q∗IQR

The parameter *q* defines the width of the range, and it can be set to a custom value according to the type of outliers we want to detect. The measurements out of the defined range are labeled as outliers. High values of *q* produce a wide range for identifying only extreme outliers.

SWAD takes in input the time series with the observations of each gas channel and performs anomaly detection on a sliding window of a predefined size (*k* measurements). Anomaly detection is performed on each channel separately. During the initialization phase of this algorithm, given an array of *k* measurements {$x_{0},\ldots ,x_{k-1}$}, mean, standard deviation, lower bound, and upper bound are calculated. When a new observation $x_{t+1}$ is received, the difference between $x_{t+1}$ and $x_{t}$ is calculated. If the variation is higher than the “difference threshold”, then $x_{t+1}$ is classified as anomalous; otherwise, the instance gets normalized using the mean and standard deviation previously calculated, as follows:

(3) $$z_{t+1}=\frac{x_{t+1}-mean}{standard\;deviation}$$

If $z_{t+1}$ is higher than the upper bound or smaller than the lower bound of the IQR range, then $x_{t+1}$ is considered an outlier. Every time a new observation $x_{t+1}$ is provided, the window is updated by removing the oldest observation $x_{t-k}$ and adding $x_{t+1}$. Finally, the parameters (mean, standard deviation, lower bound, and upper bound) are updated, and the operations to check if the data in the window are anomalies are repeated. SWAD is able to find anomalies in the measurement of each channel and gas and in temperature and humidity data.

### 4.2. Forgetting Factor Iterative Data Capture Anomaly Detection: FFIDCAD

Forgetting factor iterative data capture anomaly detection (FFIDCAD) [62] allows implementing a multivariate anomaly detection algorithm by studying the correlation of two or more correlated features. The algorithm takes as input the values of the correlated features and defines an ellipsoidal boundary around these. The observations out of bounds are classified as anomalous. We ran five algorithms: one algorithm for each pollutant and one for temperature and humidity. In our case, the correlated features for each gas were the two channels’ measurements, and for the last algorithm, the correlated features were the measurements of temperature and humidity. The first four algorithms provide the anomalies in the pollutant measurements, while the last algorithm detects anomalies in temperature and humidity.

The hyper-ellipsoid on the correlated features is defined as follows:

(4) $$ell_{k}\left(m_{k},S_{k}^{-1},t\right)=\left\{x\;in\;R^{d}\;|\;{\left(x-m_{k}\right)}^{T}S_{k}^{-1}\left(x-m_{k}\right)\leq t^{2}\right\}$$

where $m_{k}$ is the array containing the mean of the features, *x* is the data point, $t^{2}$ is the confidence space of the data distribution, and $S_{k}^{-1}$ is the inverse of the covariance that can also be defined as the precision matrix.

During the initialization phase of FFIDCAD, the mean of the first two data points is calculated, and the precision matrix *S* is initialized to an identity matrix *I* with size n × n, where *n* is the number of the analyzed features. In our case, *S* is a 2 × 2 matrix and contains the values of the working and the auxiliary channels (or temperature and humidity) of the first two consecutive observations of the time series. The diagonal elements of precision matrix measure how the variables are clustered around the mean. The off-diagonal elements measure independence, and their values are equal to 0 if features are independent. Thus, the higher the diagonal elements’ values are, the more aggregated the values are to the mean.

When a new observation $x_{k}$ is available, the precision matrix gets updated as follows:

(5) $$S_{k+1}^{-1}=\frac{kS_{k}^{-1}}{k-1}\left[I-\frac{\left(x_{k+1}-m_{k}\right){\left(x_{k+1}-m_{k}\right)}^{T}S_{k}^{-1}}{\frac{k^{2}-1}{k}+{\left(x_{k+1}-m_{k}\right)}^{T}S_{k}^{-1}\left(x_{k+1}-m_{k}\right)}\right]$$

Additionally, the mean is updated incrementally:

(6) $$m_{k}=\lambda m_{k-1}+\left(1-\lambda \right)x$$

The new instance $x_{k+1}$ is considered an anomaly if:

(7) $${\left(x_{k+1}-m_{k}\right)}^{T}S_{k}^{-1}\left(x_{k+1}-m_{k}\right)>bound$$

The bound value is calculated through the percent point function, taking the parameter *p* as the percentage. *p* is the p-value identifies the confidence space: the range of values considered non-anomalous. For example, if p=0.98, then the ellipsoid will cover 98% of the data. By assigning different values to *p*, we will obtain different confidence spaces. The closer *p* is to 1, the fewer anomalies will be found. *p* can be set through a heuristic computation, as follows: $p=1-10^{-i}$. Increasing the value of the exponent *i*, the value of *p* approaches 1, and the number of detected anomalies decreases. At the end of each iteration, the mean and precision matrix is updated considering the value of the new observation.

FFIDCAD exploits a forget factor λ∈(0,1) to update its parameters; it is introduced since after several matrix elements have been processed. The difference between $m_{k+1}$ and $m_{k}$ approaches zero, not allowing correct updating of the mean.

### 4.3. Temperature and Humidity-Based Anomaly Detection: THAD

The temperature and humidity-based anomaly detection algorithm (THAD) is based on the dependencies among all the values measured by the sensor.

Before the implementation of this algorithm, we studied the dependency between the pollutants measurements and other features: the status of the sensor, the season of the year, and the values of humidity and temperature. The aim was to find the feature that the working and the auxiliary channels are most dependent on. By analyzing our data, we found that the working and auxiliary channels’ values of NO and NO${}_{2}$ are more dependent on the temperature value, whereas O${}_{3}$ depends more on the values of humidity; therefore, the presence of anomalies was studied in relation to the value of temperature or humidity.

During the training phase of THAD, the measurements for each channel and pollutant were grouped into different ranges according to the values of temperature or humidity. The raw data collected by working and auxiliary channels of NO and NO${}_{2}$ were grouped by six predefined temperature ranges; voltages measurements of O${}_{3}$ were grouped by five predefined humidity ranges. For each group, mean and standard deviation of the pollutant measurements were calculated separately, and the lower and upper bounds were evaluated by using the IQR algorithm. There is a significant difference between this algorithm and the previous ones. Indeed, in this case, the mean, the standard deviation, and the values of lower and upper bounds are calculated only once (during the training phase), and they are not updated after the analysis of a new observation. Therefore, the anomalies of this algorithm really depend on the dataset provided for the initialization of the algorithm. Thus, the training dataset should have a comprehensive range of both temperature and humidity values, including all the seasons (one year of data).

During the detection phase, the measurement of each pollutant and channel is assigned to a group according to its value of temperature or humidity. Then, it is normalized by using the mean and standard deviation of that group. After that, the normalized value is compared to the lower bound and the upper bound of the group. If the normalized measure is out of the range, it is classified as anomalous. In this algorithm, the value of each channel is analyzed individually: if at least one channel’s value is classified as anomalous, both the measurements of that pollutant, in the corresponding observation, are considered anomalous. Anomaly detection for temperature and humidity is performed by using the IQR algorithm applied to the whole dataset. If the value of temperature or humidity is anomalous, all the measurements of that observation are classified as anomalies. However, the temperature and humidity sensors are more reliable than the other sensors (NO, NO${}_{2}$, and O${}_{3}$), and anomalies are very rare.

### 4.4. Application and Results

The anomaly detection algorithms described in the previous sections have been applied to the raw measurements collected by our 12 low-cost sensors from August 2019 to April 2021 (21 months). Each algorithm took as input the same dataset, which consists of 4,122,541 observations.

**SWAD configuration.** SWAD has been configured to use a window of 2000 observations; thus, the algorithm detects anomalies based on the data distribution of the previous 66 h, approximately. The “difference threshold” was set to 2000 to detect very different consecutive observations. After some experiments, assuming that anomalies rarely occur in our data, the parameter *q* of the IQR algorithm was set to 6, since this value showed the expected rate of anomalies (0.1%).

**THAD configuration.** The only configuration parameter used by THAD is *q*, which was set to 6.

**FFIDCAD configuration.** Some experiments were conducted to choose the exponent *i* for the parameter *p* in FFIDCAD. The value of *i* which detected the expected rate of anomalies was 16.

**Results and comparison.**Table 1 shows the number of anomalies for each gas detected by each algorithm and the ones selected by the MV. As can be seen, there are few anomalies among the values of temperature and humidity. This confirms the assumption made in the implementation of THAD. A higher number of anomalies was detected by SWAD for NO${}_{2}$ and O${}_{3}$; FFIDCAD was the one that found the least anomalies among the pollutants measurements. MV classified as anomalies on average 0.3% of the total number of measurements. By analyzing these anomalies, we noticed that the anomalies of SWAD were also detected by at least one of the other two algorithms. In particular, the percentages of anomalies detected by SWAD compared to the anomalies included in MV were 99% for NO, 100% for NO${}_{2}$, and 96% for O${}_{3}$.

### 4.5. Validation

Validating the results can be challenging, since raw air quality measurements are not human-readable and cannot be directly labeled as anomalous. We followed two different approaches: supervised evaluation with the help of environmental experts and unsupervised validation through the comparison with a well-known anomaly detection algorithm on a synthetic dataset of observations.

**Supervised evaluation considering sensor status.** Environmental experts regularly change the status of sensors when their behavior is not reliable; thus, to validate the results of the MV, we checked the status of the sensors in the timestamp of the detected anomalies. In 70% of cases, anomalies are related to “broken” status. This means that the environmental experts labeled them as unexpected behavior of the sensor, and they are likely to be real anomalies. Since we cannot know if environmental experts have recognized all the anomalies of the sensors, we still need other proofs to guarantee the validity of our methodology. For this reason, the results obtained by MV were compared with the ones obtained by the LSTM (long short-term memory) [61] autoencoder.

The **LSTM autoencoder** exploits the ability of LSTM to learn long-term dependencies. In general, autoencoders are trained to copy the input *x* to the output $\tilde{x}$. The input is compressed into a lower-dimensional domain, and the autoencoder tries to reconstruct the input from that compressed representation. To perform anomaly detection, the autoencoder has to be trained to learn the normal behavior of the sensors. Therefore, the time series taken as input by the algorithm should be anomaly-free. Then, the model is applied to the test set and allows classifying as anomalies the observations with reconstruction error greater than a pre-defined threshold. The autoencoder we used in our implementation consists of a sequence of six layers: an LSTM layer, a dropout layer, a repeat vector layer, an LSTM layer, a dropout layer, and a time-distributed layer. The first three layers are the encoder, and the last three form the decoder. The autoencoder was trained by minimizing the reconstruction error, and the used loss function was the mean absolute error (MAE). The Adam algorithm was the optimizer. Following this procedure, one model for each pollutant was generated. The number of timesteps (i.e., how many previous instances are used to predict the anomaly in the current instance) was set to 12, the number of features was 2 (i.e., the values provided by the working and auxiliary channels), and the rate of dropout was set to 0.2. For each pollutant, the training dataset consisted of 5-months data (from August 2019 to December 2019), excluding the data labeled as “broken” or “off-line” by environmental experts. For each pollutant, one model was generated and applied to the data collected from January 2020 to April 2021. For each measurement, if the difference between the reconstructed value and the real value was greater than the pre-defined threshold, the observation was classified as anomalous.

**Generation of the synthetic dataset.** In order to verify the ability of the algorithm to identify anomalies in millivolt measurements, we built a synthetic dataset. The dataset was obtained by removing from sensor observations all the measurements that were labeled as outliers by at least one of the four anomaly detection techniques described: SWAD, FFIDCAD, THAD, and LSTM autoencoder. We obtained a cleaned time series with some missing observations; thus, we replaced the missing values with the hourly average of the surrounding measurements. Finally, following the anomaly generator on time series (Agots) solution described in [63], we generated two different types of outliers: extreme outliers and variance outliers. Extreme outliers are single-point measurements that are 10 times the standard deviation from the local mean of the last 500 observations (the value can be randomly lower or higher). Variance outliers are series of adjacent observations whose differences from the surrounding measurements are 20 times higher than expected. We generated two different synthetic datasets: one with 20 extreme outliers for each channel of each pollutant, and the other with 2 variance outliers of 10 and 20 adjacent measurements for each channel of each pollutant.

**MV and LSTM autoencoder comparison.** The MV models and the LSTM autoencoder models were tested on both synthetic datasets. The results are displayed in Table 2, where the values of precision (P), recall (R), and F1-score (F1) are reported for each algorithm of MV, for the MV system, and for LSTM.

**Figure 2:**
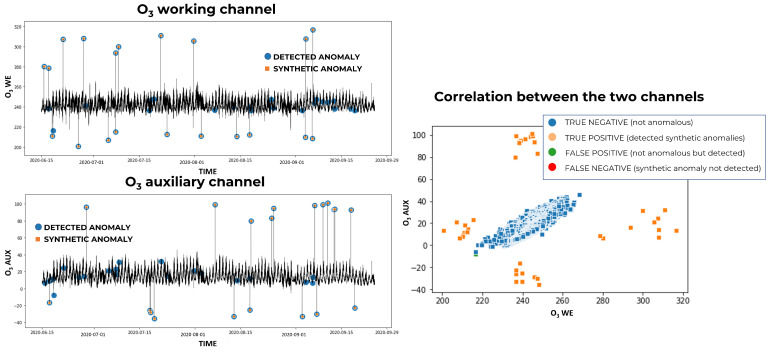
Extreme synthetic anomalies detected by FFIDCAD for O${}_{3}$.

**Figure 3:**
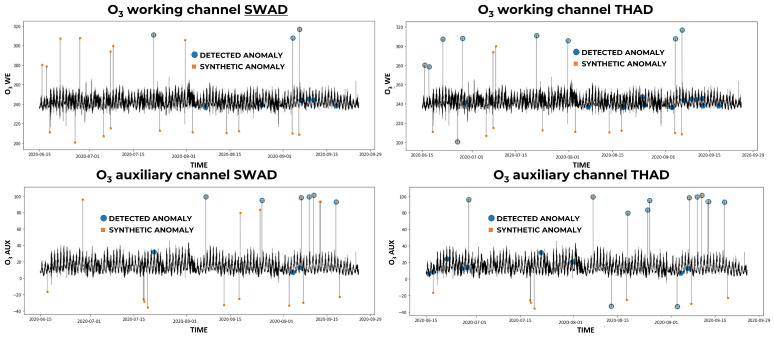
Extreme synthetic anomalies detected by SWAD and THAD for O${}_{3}$.

On the extreme synthetic dataset, FFIDCAD outperformed the other two methods. Moreover, it can be noticed that the combination of the three methods with MV always outperformed the individual models, generating a higher F1-score no matter the gas. In Figure 2, the synthetic anomalies and the anomalies detected by FFIDCAD are compared. The graphs represent the time series of the pollutant channels of the synthetic dataset, with the real extreme outliers highlighted by orange squares and the detected anomalies by blue spots. While synthetic anomalies were generated independently in each channel, our methodology works simultaneously on both channels; therefore, a synthetic anomaly in one channel does not always correspond to an anomaly in the other channel, though the anomalies detected always appear in both channels. For this reason, the two channels’ graphs need to be observed together. If a blue spot is not associated with an orange square in a channel, it may be associated with it (thus corresponding to a real extreme outlier) in the other channel’s graph. It can be observed on the right of Figure 2 that there are no false negatives (i.e., all the synthetic anomalies have been detected) and only a few false positives. As can be observed by comparing the results of FFIDCAD in Figure 2 with the results of SWAD and THAD in Figure 3, there is a major number of synthetic anomalies not bring detected by these algorithms (i.e., orange squares not corresponding to blue spots); thus, the best results are provided by the FFIDCAD algorithm, and the SWAD algorithm has the worst performances.

**Figure 4:**
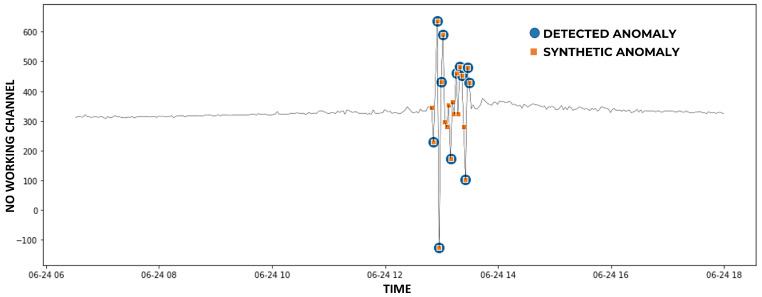
Variance synthetic anomalies detected by FFIDCAD for NO from 6 a.m to 6 p.m. of 24 June 2020.

On the variance synthetic dataset, the performances of the three methods are better than the ones obtained on the extreme synthetic dataset (considering each gas separately). However, this did not result in an increase in the performance of MV which, instead, had an F1-score lower than the one obtained on the extreme synthetic dataset. The reasons for this stemmed from the fact that the three methods found fewer coincident anomalies than in the case of the extreme dataset. Therefore, MV performed slightly worse than on the first dataset. In Figure 4, we can observe that the FFIDCAD is able to detect the majority of the variance outliers but still misses some anomalies whose values are similar to the average of the surrounding data.

Regarding MV and LSTM, the performances of the LSTM are always much worse than those of the MV, except for the variance dataset for the gas O${}_{3}$, for which the two methods are comparable. The LSTM autoencoder model failed to find the extreme outliers for NO, NO${}_{2}$, and O${}_{3}$. Although MV has a high F1-score for both NO${}_{2}$ and O${}_{3}$, the performance for NO needs to be ameliorated. In the case of variance outliers, the LSTM autoencoder still did not find any outlier for NO but had better performances for NO${}_{2}$ and O${}_{3}$. However, MV outperformed the LSTM autoencoder for all pollutants, even if the F1-score for the variance of outliers is lower than the one for extreme outliers. This is due to the fact that variance outliers are particularly challenging to detect, especially for SWAD, since they are sequences of anomalous values.

## 5. Anomaly Repairing

Once the anomalies have been detected, they are excluded from the input data of the calibration models. However, removing anomalies reduces the amount of input data and creates gaps in the time series. This strongly impacts the performances of calibration models (in our case, deep learning algorithms), which are negatively influenced by missing values. For this reason, removing anomalies is not always a good solution. Averaging data over a wider interval of time excluding anomalies can help to reduce the number of missing values. However, aggregating values often means supposing that the missed values are equal to the average of the values in the same time interval. This approximation does not always fit the use case. In addition, if in the time interval, all the raw measurements are labeled as anomalies, some gaps still remain in the aggregated time series.

In this section, the AIrSense methodology for anomaly repairing will be described. The proposed solution is suitable for real-time applications, since it is based only on past data and has a short execution time. Firstly, raw measurements are aggregated over a given time interval (10 min, in our use case), while excluding anomalies. Then, the gaps are repaired by forecasting the value with a vector autoregression (VAR) model trained on the reliable previous raw observations, as described in Section 5.1. Finally, the predicted value is substituted into the time series and used to forecast and repair future gaps in the data sequence. This is repeated iteratively for each missing value (as described in Section 5.2). The described methodology was applied to raw measurements coming from low-cost AQ sensors. The results are discussed in Section 5.3.

### 5.1. VAR Model

Sensor raw observations are comprise a collection of values for each time instant, which generates a multivariate time series. In the case of AQ sensors, the measured values are correlated, since chemical cells are influenced by the presence of other pollutants and weather conditions (temperature and humidity). VAR is a statistical model for the simultaneously forecasting of all the variables in a multivariate time series. The time series is modeled as a linear combination of its own past values. In the case of multivariate time series, each variable forecast is evaluated considering its previous values and the values of the other variables in the previous time instants [64]. Given a multivariate time series *Y* with *K* variables that is composed of *T* observations, its values at the instant *t* is evaluated as:

(8) $$Y_{t}=\beta +\alpha_{1}Y_{t-1}+....+\alpha_{p}Y_{t-p}+\varepsilon_{t}$$

where β is a K × 1 intercept vector that depends on the season associated with *t*; $\alpha_{1}$ to $\alpha_{p}$ are the coefficients of the previous lags of *Y* till order *p*. $Y_{t-1}$ to $Y_{t-p}$ are the 1 × K vectors representing the *p* lags of *Y* used to predict the actual value of $Y_{t}$. $\varepsilon_{t}$ is the normally distributed error. Thus, the multivariate time series is modeled as a system of equations with one equation per variable.

**Verification of the assumptions.** The VAR model is based on the assumption that each variable in the time series is influenced by the others. Therefore, we executed the Granger Causality test [65,66] on our data and verified this condition. The Granger causality test is a statistical hypothesis test for determining whether one time series is useful for forecasting another. A variable $v_{1}$ Granger-causes a variable $v_{2}$ if the past values of $v_{1}$ add power to forecasting the actual value of $v_{2}$ after considering the past values of $v_{2}$ [67]. The Granger causality tests the null hypothesis that the coefficients of the past values of the variable $v_{1}$ in the regression equation are zeros. If the null hypothesis is neglected, then $v_{1}$ causes $v_{2}$. The test is repeated between each couple of variables. Moreover, to apply the VAR model, time series should be stationary. We employed the augmented Dickey–Fuller (ADF) [68] test to verify the stationarity of each time series. In a multivariate time series, the time series associated with each variable is tested separately from the others to determine its stationarity. However, if even only one variable is not-stationary, all the variables must be differentiated. This is because differencing a single time series reduces its length for that variable, losing the correspondence with the others.

**Parameter evaluation.** Finally, the VAR model can be applied to the multivariate time series itself or a differentiated version. The value of *p* is evaluated considering the value that generates a model with the lowest Akaike information criterion (AIC). The AIC evaluates the quantity of lost information when the model is used to describe reality [69]. When the time series is differentiated, the forecast of the VAR model needs to be inversely transformed, adding to the forecast the values of the last element of the time series.

### 5.2. The Iterative Repairing Procedure

**Figure 5:**
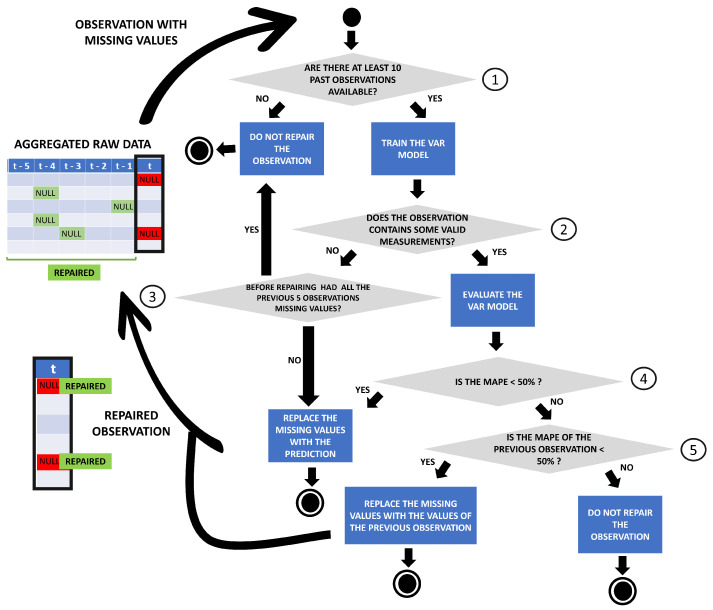
The repairing procedure applied on each raw observation with missing values and restarted when the sensor status change.

The repairing procedure needs to be applied in real-time; therefore, in order to repair missing values in a real-time data stream, we can only rely on past measurements. There are two different types of missing values we need to deal with: the ones caused by the inability of the sensor to measure a variable or communicate its value, and the gaps caused by the presence of anomalies. The repairing procedure tries to repair both types of missing values, but the first type may cause a long series of gaps for consecutive observations. The repairing procedure is described in Figure 5.

**VAR model fitting.** For each raw observation with missing values, the past observations are taken into account to predict the value that is missing. As can be seen in point 1 in Figure 5, the number of previous observations without missing values in similar conditions (e.g., same position, same environment and same sensor status) must be higher than a threshold (e.g., 10 observations), or the observation is not repaired. Then, all the previous observations in similar conditions are used to fit a VAR model and predict the value of each variable. Before fitting the model, the stationarity of the time series is verified, and if necessary, the time series is differentiated a first and a second time. If after two differentiations, the time series is still not stationary, the missing value is not repaired (point 2 in Figure 5), and if in all the previous five observations there was a missing value, the observation is not repaired. If in the previous five observations there was at least one observation without missing values, the trained VAR model is used to predict the value, and the missing value is replaced with the prediction (point 3 in Figure 5).

**Evaluating the performance of the fitted model.** When we obtained the VAR prediction, we tried to evaluate the performance of the fitted model on the variables that were measured by the sensor. When a value is missing for one or more variables, this does not mean that all the observed variables are missing. If some variables in the missing observation have an associated value, we can measure the error of the VAR forecast value for that variable. The mean absolute percentage error (MAPE) was evaluated for all the available variables. Since we had only one observation, the MAPE was evaluated as:

$$MAPE=100\cdot \left|\frac{F-R}{R}\right|$$

where *F* is the forecasted value and *R* the real value. A value of MAPE is obtained for each variable; MAPEs are then averaged, and if the mean value is above 50%, the forecast is considered not reliable (point 4 in Figure 5). Thus, the VAR model was not able to correctly predict the other variables, and we assume that the prediction for the missing variable is not trustworthy. In this case, an additional attempt to predict and repair the value is conducted. The previous observation before the one to repair is taken into account as a possible prediction. Therefore, as can be seen in point 5 in Figure 5, the MAPE error between the available values of the observation to repair (considered as real values) and their values in the previous observation is evaluated. If the average of these MAPE errors is below 50%, then the observation is repaired by replacing the missing values with the values of the previous observation. When all the variables of the observation to repair are missing, no evaluation can be performed; thus, if the number of previous observations in which all variables are missing is higher than five, we do not repair the observation. Repaired observations should be reliable; this conservative approach avoids generating unreliable predictions.

**Repeating the process.** Each repaired observation is used to predict the values of subsequent gaps in the multivariate time series in an iterative process. For each gap, a new VAR model is fitted while considering the previously repaired observations. Thus, in the case of consecutive gaps in the time series, we will have to rely on fully repaired observations to predict all variables and we risk producing unrealistic observations. The iterative process is repeated for each sensor’s status, so when the sensor is moved or its status changes, the iterative process is restarted.

### 5.3. Application and Results

From August 2019 till September 2020, the raw data of the 12 sensors reported 12,567 anomalies that generated 262 missing values in the 10-min aggregated data. Not all these gaps can be repaired with the described process, since some of them are consecutive and the repaired values are not reliable. Exploiting the results of the Granger Causality test, we decided to remove temperature and humidity from the creation of the VAR model. The repairing procedure solved 177 gaps, reducing the number of missing values to 85. 133 out of 177 gaps are repaired using VAR model, since its prediction has an average MAPE error less than 50% (12% on the average). The remaining 44 gaps were repaired with the values of the previous observation for that variables, and their average MAPE was 26%. The repairing procedure is very fast and requires around 1600 microseconds for each observation that needs to be repaired.

## 6. Experiments and Evaluation

**Figure 6:**
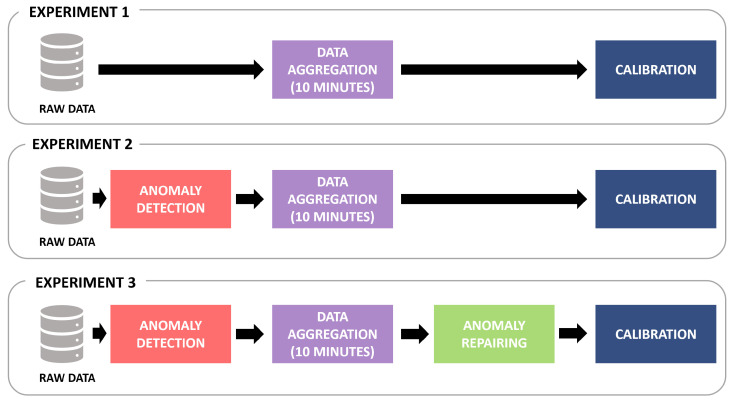
Description of the data flow in the proposed experiments.

To evaluate the effects of anomaly detection and repairing on the calibration models (LSTM) that estimate the pollutant concentration values, three different experiments were conducted on the raw data collected from the low-cost sensor network of the city of Modena for NO, NO${}_{2}$, and O${}_{3}$. Figure 6 displays and compares the data flow of the experiments. The reproducibility is guaranteed, since all datasets are available as open data (https://drive.google.com/drive/folders/1LqZSVXA_2A1Hk_7fk9UwDOYEda-J6qvG (accessed on 30 December 2022)).

**Exp1**: The 10 min of aggregated raw data were directly used to train the calibration models. Then, in the test phase, raw data were directly aggregated without excluding anomalies and then given as input to the calibration models.

**Exp2**: The MV anomaly detection methodology described in Section 4 was employed to recognize anomalies in raw data. Then, only the reliable raw measurements were used to evaluate a 10 min average and generate the aggregated time series used as input for the calibration models.

**Exp3**: After applying the MV methodology to detect anomalies and after aggregating only the reliable data every 10 min, the missing values were repaired using the VAR-based iterative process described in Section 5. Finally, the repaired aggregated time series was used to fit the calibration models and test them.

The training dataset of the three experiments contains data from August 2019 to March 2020, whereass the test dataset includes data from the 15th of June to the end of September 2020. To train and test the models, we considered only the period in which the sensor was located near the legal station (“calibration” mode). As described in Section 3, the calibration was performed through an LSTM model. In order to evaluate the performances of the calibration models, the concentrations of pollutants generated by applying the models in the test dataset were compared to the measurements of the legal station.

### 6.1. Evaluation Metrics

The metrics used to evaluate the results are root mean-square error (RMSE) and accuracy. RMSE is the root of the mean of the squared differences between calibrated values and corresponding values observed by the legal station in the same 10 min time interval. Since the differences are squared before they are averaged, the RMSE penalizes large errors. RMSE can range from 0 to *∞*. RMSE values give an idea of the absolute value of the error on the calibrated values. In some applications that use a range of values to derive information about air quality, these errors are immaterial. This is the case for air quality dashboards, where AQ information is conveyed through color scale maps. Several color scales with different ranges are available. We used the one provided by the European Environmental Agency (EEA) (https://www.eea.europa.eu/it (accessed on 30 December 2022)) to measure the ability of our algorithms to correctly predict the right color class. Both observed values from the legal station and our calibrated values were associated with the corresponding color in the color scale. We calculated the accuracy as the ratio between the number of correct predictions and the total number of input samples.

Table 3 shows the values of RMSE and accuracy obtained for each gas and each sensor in the three experiments. In addition, for each gas and experiment, average values of RMSE and accuracy are provided. On average, the RMSE values of **Exp3** are always lower than the ones of **Exp1** and **Exp2**. In **Exp2**, the number of anomalies detected in the training set was 4,198, whereas for the test set it was 700. The effect on the performance of the model was a significant reduction in the RMSE. However, the exclusion of anomalies generated 38 missing values in the multivariate time series during the test phase of **Exp2**, and for the corresponding time instants, the calibrated values were not generated. In **Exp3**, 30 out of 38 missing values in the test data and 128 out of 151 in the training data were repaired. As a consequence, the RMSE error reduced by 3.12 on an average compared with **Exp2** and by 29.05 compared with **Exp1**.

### 6.2. Result and Discussion

For all the pollutants, the RMSE was significantly reduced after introducing anomaly detection before calibration. Moreover, for NO${}_{2}$ and O${}_{3}$, adding the repairing procedure further decreased RMSE and improved accuracy. A particular case was the one of sensor 4008 that reported a very high RMSE values in **Exp1** for both NO and NO${}_{2}$. By analyzing the difference between our calibrated data and the actual concentrations provided by the legal station, we noticed that there is a very large difference in only one 10-min interval, and this difference strongly influenced the value of the RMSE. Some raw measurements related to that 10-min interval were classified as anomalies in **Exp2**, so the aggregated value changed and the RMSE decreased. This value decreased again in **Exp3** as a consequence of repairing the anomalous data in raw measurements.

As can be seen in the table, the values of accuracy are always very high for NO and NO${}_{2}$. O${}_{3}$ has lower values of accuracy. This could have two explanations. Firstly, the size of the training set for O${}_{3}$ is smaller than the ones for NO and NO${}_{2}$, since O${}_{3}$ is measured by only one legal station. Therefore, the sensors have to be close to that legal station to collect data useful for the calibration. Secondly, the ranges of the color scale for O${}_{3}$ are 20 units in size, whereas the ones for NO and NO${}_{2}$ are 50, 100, and 200 units in size. Therefore, a small error in O${}_{3}$ affects the classification.

## 7. Conclusions

This paper, to the best of our knowledge, is the first research effort to address anomaly detection and repairing on raw air quality data that considers the temporal sequence of the measurements and exploits the correlations between various sensor features. This approach aims to improve the calibration performance on multivariate time series. In the literature, anomaly detection and repairing methodologies have usually been applied as post-processing techniques on the calibrated AQ observations. However, as demonstrated by comparing the results of **Exp1** and **Exp2**, removing anomalies from raw data reduces the error in the calibrated values. We defined an iterative procedure to repair missing values that can be applied in real-time to a data stream to increase the coverage of AQ data. The calibration algorithm trained on the repaired data (**Exp3**) had better performances, demonstrating the importance of a combined approach for anomaly detection and repairing in data-driven models.

The AIrSense framework, which we have proposed in this paper, has been proved to be a robust and effective solution for performing real-time AQ monitoring. It has been extensively used in the city of Modena and can be easily adapted to different contexts. We have worked on the amelioration of the actual methodology in order to improve the performance. In particular, for the experiments performed in this paper, the “difference threshold” of the SWAD algorithm was a hyperparameter that was fixed to a given value (https://drive.google.com/drive/folders/1LqZSVXA_2A1Hk_7fk9UwDOYEda-J6qvG (accessed on 30 December 2022)). However, this is not the best solution, and a better approach would be to adapt and evaluate this threshold based on the values of each window. We have already developed an updated version of the framework that implements this new solution (https://github.com/ChiaraBachechi/AQAnomalyDetectionFramework (accessed on 30 December 2022)).

## Figures and Tables

**Table 1:** Anomalies detected by SWAD, FFIDCAD, and THAD.

	NO	NO${}_{2}$	O${}_{3}$	Temperature	Humidity
SWAD	16,720	35,436	38,986	1176	1176
FFIDCAD	6577	4705	5364	562	562
THAD	9050	14,606	26,978	0	0
**MV** (% of total)	**10,591** (0.26%)	**15,365** (0.37%)	**17,365** (0.42%)	**9** (~0%)	**9** (~0%)

**Table 2:** Performance evaluation on synthetic datasets.

		NO					NO${}_{2}$					O${}_{3}$				
		SWAD	FFIDCAD	THAD	MV	LSTM	SWAD	FFIDCAD	THAD	MV	LSTM	SWAD	FFIDCAD	THAD	MV	LSTM
**Extreme**	**R**	0.15	0.3	0.2	0.42	0	0.34	0.64	0.25	0.95	0	0.14	0.5	0.3	0.97	0
	**P**	1	0.34	1	0.33	0.68	1	0.83	1	0.81	0	1	0.71	1	0.97	0
	**F1**	0.26	0.32	0.34	0.37	0	0.5	0.72	0.4	0.87	0	0.24	0.59	0.46	0.97	0
**Variance**	**R**	0.25	0.4	0.3	0.3	0	0.35	0.98	0.4	0.65	0.38	0.23	1	0.55	0.5	0.53
	**P**	1	0.31	1	0.35	0.82	1	0.81	1	0.83	0.82	1	0.98	1	0.73	0.68
	**F1**	0.4	0.35	0.46	0.32	0	0.52	0.89	0.57	0.73	0.52	0.37	0.99	0.71	0.59	0.6

**Table 3:** Experimental evaluation.

		RMSE			ACCURACY		
Gas	Sensor	Exp1	Exp2	Exp3	Exp1	Exp2	Exp3
**NO**	4003	5.24	3.53	**3.18**	0.99	0.99	0.99
	4005	2.82	**2.34**	2.94	1	1	1
	4006	**2.53**	2.59	2.71	1	1	1
	4007	**2.74**	3.15	4.5	0.99	0.99	0.99
	4008	93.24	4.37	**2.62**	0.99	0.99	1
	4010	3.8	**2.48**	5.18	0.99	0.99	0.99
	4011	4.16	**3.92**	4.23	0.99	0.99	0.99
	4013	26.24	**2.2**	2.73	0.99	0.99	0.99
	4014	2.39	2.81	**2.05**	1	1	1
	**M**	**15.91**	**3.04**	**3.35**	**0.99**	**0.99**	**0.99**
**NO${}_{2}$**	4003	8.25	15.07	**6.53**	0.97	0.98	0.98
	4005	**12.2**	12.44	12.44	0.95	0.95	0.95
	4006	9.55	**8.81**	9.09	0.97	0.97	0.97
	4007	10.76	9.83	11.89	0.95	0.95	0.95
	4008	347.03	9.38	**6.62**	0.96	0.96	0.97
	4010	7.78	7.58	**7.11**	0.98	0.98	0.98
	4011	64.54	8.89	**8.58**	0.98	0.98	0.98
	4013	8.01	8.2	**7.96**	0.98	0.98	0.98
	4014	10.05	9.33	**7.82**	0.96	0.97	0.96
	**M**	**53.13**	**9.95**	**8.67**	**0.97**	**0.97**	**0.97**
**O${}_{3}$**	4003	20.03	18.51	**18.05**	0.53	0.56	**0.58**
	4005	19.9	**17.7**	18.78	0.65	**0.75**	0.73
	4006	19.41	**17.67**	17.87	0.61	**0.65**	**0.65**
	4007	14.62	**12.23**	13.41	0.8	0.82	**0.85**
	4008	**19.79**	22.49	22.66	**0.61**	0.57	0.57
	4013	25	**22.52**	22.68	0.48	0.5	**0.54**
	4014	222.53	86.27	**14.64**	**0.71**	0.52	0.66
	**M**	**48.75**	**28.20**	**18.30**	**0.63**	**0.62**	**0.65**

## Data Availability

The code of the AIrSense framework and the data used in the experiments, both real-world data and the synthetic dataset, are publicly available here: (https://drive.google.com/drive/folders/1LqZSVXA_2A1Hk_7fk9UwDOYEda-J6qvG (accessed on 30 December 2022)). Moreover, the raw and calibrated data are available as open data on the Emilia Romagna regional data portal (https://dati.emilia-romagna.it/ (accessed on 30 December 2022)) and on the National and European data portals; the hourly data of the legal stations are available on the ARPAE data portal (https://dati.arpae.it/dataset/qualita-dell-aria-rete-di-monitoraggio (accessed on 30 December 2022)).
